# ZNF703 Overexpression may act as an oncogene in non‐small cell lung cancer

**DOI:** 10.1002/cam4.847

**Published:** 2016-09-20

**Authors:** Onur Baykara, Nejat Dalay, Kamil Kaynak, Nur Buyru

**Affiliations:** ^1^ Department of Medical BiologyIstanbul University Cerrahpasa Faculty of MedicineIstanbulTurkey; ^2^ Istanbul University Oncology InstituteIstanbulTurkey; ^3^ Department of Chest SurgeryIstanbul University Cerrahpasa Faculty of MedicineIstanbulTurkey

**Keywords:** Akt/mTOR pathway, expression, NSCLC, ZNF703

## Abstract

Despite therapeutic advances, lung cancer remains one of the most common causes of cancer‐related deaths worldwide. The ZNF703 gene has been identified as the driver of the 8p11‐12 region and its amplification or overexpression has been associated with several types of cancers. It has also been shown that ZNF703 overexpression can activate the Akt/mTOR signaling pathway. The aim of our study was to investigate the role of the ZNF703 gene in association with Akt/mTOR activation in non‐small cell lung cancer (NSCLC). Expression levels in tumors and matched noncancerous tissue samples from 47 patients were analyzed by qRT‐PCR and the Akt phosphorylation levels were investigated by Western blotting. Our results show that ZNF703 is up‐regulated in 63.4% of NSCLC tumor samples. Althogh the correlation did not reach a statistically significant level Akt phosphorylation was increased in tumor tissues expressing high levels of ZNF703. The role of the ZNF703 gene has not been investigated in NSCLC. Our data show that ZNF703 may contribute to tumor development in NSCLC by activating the Akt/mTOR pathway.

## Introduction

Epidemiological studies indicate tobacco smoking and alcohol consumption as one of the main causes for lung cancer 1. However, cytogenetic studies using comparative genomic hybridization (CGH) and fluorescence in situ hybridization (FISH) have already associated chromosomal aberrations with non‐small cell lung cancer (NSCLC) 2, 3. Chromosomal aberrations include the loss or gains of partial or whole chromosomal arms on several chromosomes and are a hallmark of cancer cells. Increases in the gene copy numbers due to chromosomal amplifications constitute a common mechanism for oncogene activation. In particular, oncogene activation through increased gene copy numbers resulting in overexpression contributes to the malignant transformation in various solid tumors, including NSCLC.

Recurrent genetic alterations at chromosome 8p11‐12 have been associated with advanced disease and poor patient outcome in several types of malignant diseases 4. This was suggestive of presence of oncogenes in this region and numerous studies have been carried out to identify these genes. As a result of these studies several genes such as ZNF703, SPFH2/ERLIN2, BRF2, PROSC, and RAB11F1P1 within the 8p11‐12 chromosome region have been identified as candidate oncogenes 5, 6. Among these ZNF703 has been characterized as the genetic driver of the 8p12 amplicon in luminal B breast cancer 7, 8, 9, 10, 11.

ZNF703 is a member of the NET/NLZ family of transcription factors 12. NET family proteins have crucial functions in a variety of developmental processes 12, 13. In humans two NET family members, NLZ1/ZNF703 and NLZ2/ZNF503 have been identified. NLZ1/ZNF703 is ubiquitously expressed in human tissues and localizes to the nucleus under nonpathological conditions 14. Even though its exact function is unknown it has been proposed that its function depends on cellular context. However, it has been shown that the ZNF703 gene is amplified and/or overexpressed in breast, colorectal, and gastric cancers 15, 16, 17. The gene is also reported to be involved in the activation of the Akt/mTOR signaling pathway in breast cancer cell lines 18. The Akt1 protein can be activated by different pathways and phosphorylation at the Ser473 residue is an indicator of its activation. A recent study demonstrated that enhanced ZNF703 expression represses E‐cadherin expression and increases lung metastasis rates in a mouse model of breast cancer 8.

In our previous study, we identified amplification of the ZNF703 in 46.3% of the NSCLC tissues by MLPA analysis 19. Depending on these results we proposed that ZNF703 may also play an important role in the pathogenesis of NSCLC. To our knowledge there is no report in the literature investigating ZNF703 expression in NSCLC. To test a probable activating effect of ZNF703 on the Akt/mTOR pathway we also investigated the phosphorylation levels of the Akt protein in the same cohort.

## Methods

Tumor and the corresponding normal lung tissue samples were obtained from 47 patients with NSCLC undergoing surgery at the Istanbul University Cerrahpasa Medical Faculty, Department of Chest Surgery. The patients taken into the study had not received any previous therapy and were admitted to the hospital for the removal of the tumor as the primary treatment. The study was approved by the Ethical Committee of the Istanbul Faculty of Medicine (292/06.02.2014) and signed informed consent was obtained from all patients.

### DNA isolation

DNA was isolated using the Roche Diagnostics DNA isolation kit (Roche Diagnostics, Mannheim, Germany) and gene copy numbers were determined by multiplex ligation‐dependent probe amplification (MLPA) using the ABI PRISM 3100 genetic analyzer (Applied Biosystems, Foster City, CA) as reported previously 19. Briefly, peak areas of the amplification products were normalized using the Genemapper program (Applied Biosystems) and the data evaluated by comparing with the peaks from healthy tissue using the Coffalyzer analysis software (MRC‐Holland, Amsterdam, the Netherlands) to determine the gene copy numbers. Values below 0.75 were defined as losses and values higher than 1.25 were defined as amplifications.

### RT‐PCR and real‐time quantitative RT‐PCR

Total RNA from the tissues was isolated by using the PureLink RNA Mini Kit (Ambion, Thermo Fisher Sci., Carlsbad, CA, USA) according to the manufacturer's instructions. cDNAs were prepared from 400 ng of total RNA using the Transcriptor First Strand cDNA Synthesis Kit (Roche Diagnostics). Expression level of the ZNF703 gene in the tumors and noncancerous tissue samples were analyzed by Quantitative Real Time PCR (qRT‐PCR) using the LightCycler 480 system (Roche Diagnostics). PCR reactions were performed in a final volume of 20 *μ*L containing 1 × Master Mix, 300 nmol/L gene specific primers (forward: 5′‐ GTCCTCCACTCCCGTCAG‐ 3′ and reverse: 5′‐ CCACCGAGTTGAGTTTGGAG‐3′) and 200 nmol/L hydrolysis probe (UPL Probe No.70) for ZNF703 which was labeled with fluorescein (FAM) at the 5′‐end and with TAMRA at the 3′‐end. The Glucose‐6‐Phosphate Dehydrogenase (G6PD) gene was used as the reference to normalize the mRNA levels of each sample for quantification. (Primers; forward: 5′‐ CATGGTGCTGAGATTTGCCAAC‐3′ and reverse: 5′‐ TCAACACCTTGACCTTCTCATCAC‐3′) probe 5′‐FAM‐ATCCGGGACGTGATGCAGAACCACCTAC‐TAMRA‐3′). Relative mRNA levels were determined by comparing the expression level of each tumor sample to its matched normal counterpart by using the 2^−ΔΔCt^ method 20. Values between 0.9 and 1.1 were considered as no change, and values below and beyond these were defined as “decreased” and “increased”, respectively.

### Western blotting

Fresh tumor samples were taken during surgery and were immediately processed in the laboratory without freezing. Cells obtained from the cancerous and normal lung tissues were lysed in ice‐cold RIPA (radioimmunprecipitation assay) buffer (25 mmol/L Tris HCl, pH 7.6, 150 mmol/L NaCl, 1% NP‐40, 1% sodium deoxycholate, 0.1% SDS), with 1% sodium orthovanadate, 1% phenylmethylsulfonyl fluoride (PMSF) and 1% protease inhibitor cocktail to obtain the whole cell lysate. Protein aliquots (30 *μ*g each) were resolved on 12% SDS‐PAGE gels and transferred to nitrocellulose membranes (Life Technologies, Carlsbad, CA) using the iBlot Dry Blotting System (Life Technologies). After washing, the membranes were incubated with 5% Blocking Solution (Tris‐buffered saline/Tween 20‐nonfat dry milk), overnight at 4°C and probed with the 1:200 diluted primary antibodies Akt1 (sc‐5298‐Santa Cruz Biotechnology, Dallas, TX) or p‐Akt1 (Ser473) (sc‐293125‐Santa Cruz Biotechnology). After washing three times with 1xTBST for 5 min, the membranes were incubated for 1.5 h at room temperature with the 1:1000 diluted HRP‐conjugated secondary antibody (Goat anti‐mouse IgG‐HRP (sc‐2005‐ Santa Cruz Biotechnology). After washing five times with 1xTBST for 5 min, the protein bands were detected using the Western Lightening Chemiluminescence Reagent (Life Technologies) with an exposure time of 7 min. The intensity of luminescence was quantified with the Image J 1.47 software bundled with the Java analysis program. The ratio of the “phosphorylated Akt/Total Akt” was determined for each individual sample by calculating the areas of the corresponding bands (Table S1).

### Statistical analysis

Statistical analysis was performed using the SPSS 21 for Windows (IBM Corp. Released version 2012, IBM SPSS Statistics for Windows, Version 21.0. Armonk, NY: IBM Corp.). We used the *χ*2 (2‐tailed) test to analyze the association between gene expression levels and copy numbers or clinicopathological characteristics and the Spearman's Rho test to evaluate the correlation between the expression level of ZNF703 and Akt phosphorylation. *P* < 0.05 was considered statistically significant.

## Results

ZNF703 is located on chromosome 8p11‐12 where gene amplification has frequently been observed in human tumors, including the lung. In our previous study we identified the ZNF703 gene as the most frequently amplified gene (38/82, 46.3%) in that region of the NSCLC tumors samples 19. Therefore, to identify whether this amplification results in overexpression of the gene we used qRT‐PCR to determine the ZNF703 expression levels in 47 NSCLC tumors and matched noncancerous tissue samples. We observed ZNF703 overexpression in a high fraction of tumor tissues (63.4%) compared to the noncancerous tissue samples. However, this overexpression was not associated with copy number increase (Table 1). In five of the tumor tissues and 10 of the noncancerous tissues ZNF703 mRNA was not present. The average increase in ZNF703 mRNA in the tumor tissues was 55% when compared with corresponding normal tissues (Table 2). When we evaluated the association between ZNF703 gene expression in the tumors and clinicopathological data we found no correlation between the levels of ZNF703 expression and the clinicopathological characteristics of the patients (Table 3).

**Table 1:** Association between ZNF703 expression, p‐Akt and ZNF703 copy numbers

ZNF703 expression	MLPA copy number of ZNF703 *n* (%)				p‐Akt n (%)		
	Increased	Decreased	No change	*P*	Increased	Decreased	*P*
Increased	9 (21.4)	–	17 (40.5)	0.41	15 (35.7)	11 (26.2)	0.78
Decreased	5 (11.9)	1 (2.4)	9 (21.4)		8 (19.1)	7 (16.6)	
No change	–	–	1 (2.4)		–	1 (2.4)	

ZNF703 and p‐Akt expression were determined by quantitative RT‐PCR and Western Blotting respectively. Copy number variations were evaluated by MLPA. MLPA, multiplex ligation‐dependent probe amplification.

**Table 2:** Expression levels of the ZNF703 gene in the samples

	ZNF703 C_t_ (Mean)	G6PD C_t_ (Mean)	∆C_t_	∆∆C_t_	2^−∆∆Ct^
Tumor	28.39	26.92	1.47	−0.63	1.55
Normal	31.34	29.24	2.1	−	−

**Table 3:** Association of ZNF703 expression and Akt phosphorylation with the clinical parameters

	ZNF703 Expression increased	ZNF703 Expression decreased	No change	*P*	p‐Akt increased	p‐Akt decreased	*P*
Pathology				0.837			0.144
Adeno	11 (26.2)	5 (11.9)	1 (2.4)		7 (16.7)	10 (23.8)	
SCC	11 (26.2)	7 (16.7)	–		13 (30.1)	5 (11.9)	
Others	4 (9.5)	3 (7.1)	–		3 (7.1)	4 (9.5)	
Stage				0.85			0.389
1‐2A	14 (33.4)	8 (19.1)	1 (2.4)		14 (33.4)	9 (21.4)	
2B‐4	10 (23.8)	5 (11.9)	–		7 (16.7)	8 (19.1)	
n/a	2 (4.7)	2 (4.7)	–		2 (4.7)	2 (4.7)	
Gender				0.903			0.667
Male	24 (57.1)	14 (33.4)	1 (2.4)		21 (50)	18 (42.8)	
Female	2 (4.7)	1 (2.4)	–		2 (4.7)	1 (2.4)	
Age				0.629			0.488
<50	5 (11.9)	2 (4.7)	–		3 (7.1)	4 (9.5)	
>50	21 (50)	13 (30.1)	1 (2.4)		20 (47.6)	15 (35.8)	
Smoking				0.218			0.581
30 pk/year	14 (33.4)	5 (11.9)	–		12 (28.6)	7 (16.7)	
31–60 pk/year	8 (19.1)	8 (19.1)	1 (2.4)		8 (19.1)	9 (21.4)	
>60 pk/year	1 (2.4)	2 (4.7)	–		2 (4.7)	1 (2.4)	
n/a	3 (7.1)	–	–		1 (2.4)	2 (4.7)	

Adeno, Adenocarcinoma, SCC, Squamous cell carcinoma. Others: large cell and adenosquamous carcinomas. pk/year: packs/year

We also investigated phosphorylation of the Akt1 protein as an indicator of the Akt1/mTOR pathway activation in 43 tumor samples in association with ZNF703 expression. An increase in Akt1 phosphorylation was observed in 54.7% of the tumor tissue samples. The increase in p‐Akt1 was more frequent in the samples displaying ZNF703 overexpression. However, this positive association was not statistically significant probably due to the low number of samples (*P* = 0.7) (Table 1).

## Discussion

Chromosomal amplifications are critical molecular events which are observed in the pathogenesis of lung cancer. Recurrent gains of partial or whole chromosomal arms on 1q, 3q, 5p, 8p, and 11q have been reported in NSCLC 2, 3, 21. Oncogene activation, through gene activation resulting in overexpression, contributes to malignant transformation in many solid tumors. Association of the amplification of the chromosome 8p11‐12 region with human breast cancer suggested that this region contains tumor/metastasis‐promoting genes 22. As a result of investigations to identify the genes which lie in that region, ZNF703 has been identified as the driver oncogene within this region 5, 11. Successive studies have shown that the ZNF703 gene can act as an oncogene in luminal B breast cancers 7, 9, 11. It has also been shown that ZNF703 is overexpressed in 15% of breast cancers that harbor 8p12 amplifications 23, 24. In our previous study we detected ZNF703 amplification in 43.2% of the NSCLC tumor tissues 19. Therefore, in this study we investigated the expression of ZNF703 to identify whether overexpression of ZNF703 occurs as a result of amplification in NSCLC. To our knowledge, there is no study in the literature analyzing the ZNF703 gene in the pathogenesis of NSCLC. However, overexpression of ZNF703 has been reported in colon, gastric and head and neck squamous cell cancers which indicates that ZNF703 acts as an oncogene in different kinds of cancer in addition to breast tumors 15, 16, 17, 18, 25. In our study group we observed ZNF703 overexpression in 64.1% of NSCLC tumor samples harboring ZNF703 amplification 19. This frequency was higher than in breast cancer. However, in almost all of these studies the protein has been analyzed by immunohistochemistry or Western blotting. In only one of these reports ZNF703 expression has been investigated quantitatively at the mRNA level using qRT‐PCR in a small group of patients with colon cancer 18. In accordance with our results they have found that the ZNF703 gene is overexpressed in 72.7% (16/22) of the colorectal tumor tissues. High ZNF703 expression has been shown to activate E2F1 transcription driving proliferation of the tumors 9. Lack of association between gene amplification and ZNF703 expression in the present study is most likely due to the heterogeneity of the tumors and is not unexpected since a positive correlation between these parameters has not been observed consistently 15. Furthermore, copy number alterations may not be the only mechanism by which ZNF703 is deregulated. ZNF703 may act as an oncogene in only a subset of the tumors as has been reported for breast cancer 7. Supporting this possibility analysis of the cBIO portal (http://www.cbioportal.org/index.do) for cancer genomics has shown that copy number changes of the ZNF703 gene are much higher in the squamous cell carcinomas of the lung when compared to the adenocarcinomas 26, 27. In the present study we did not find a correlation between the mRNA levels and clinicopathological factors. However, this fact does not exclude the possibility that a correlation between the clinical factors and protein levels may still exist since the proteins are the functional units in the cells.

Despite the growing interest in ZNF703 its exact role in the pathological processes and its mechanism of action are not known. Slorach et al. 8 have shown that overexpression of the mouse ortholog of ZNF703, zeppo1 (Zpo1) regulates MEC adhesion, migration and polarity. Additionally they also reported that Zpo1 is a driver of metastasis in breast carcinogenesis. Depending on these results they proposed that Zpo1 promotes metastasis via repressing E‐cadherin expression. More recently, overexpression of ZNF703 in MCF‐7 breast cancer cell lines has been associated with the activation of the Akt/mTOR signaling pathway 18. The Akt/mTOR pathway is one of the critical pathways in tumorigenesis and phosphorylation of Akt at Ser473 is an indicator of the activation of this pathway. Therefore, to test this hypothesis we investigated Akt phosphorylation levels in tumor tissues in association with ZNF703 expression. Supporting an association between ZNF703 and Akt1 phosphorylation our results show that Akt phophorylation is increased in 65.2% (15/23) of ZNF703‐over‐expressing NSCLC tumors and indicate that by the overexpression of ZNF703 one of the most critical tumorigenic pathways is activated. A possible role of ZNF703 in inducing production of growth factors or stimulating the growth factor pathways has been suggested previously 15. A recent report showing elevated Focal Adhesion Kinase (FAK) activity in tumors driven by the closely related ZNF503 protein 28 supports our data for the role of ZNF703 in the stimulation of the Akt pathway since FAK is directly regulated by Akt 29. In view of the importance of FAK in promoting cell survival and proliferation and its upregulation as well as involvement in the progression of several tumors a possible interaction between ZNF703 and FAK remains to be elucidated 30, 31.

## Conclusions

Our study is the first report in the literature investigating the expression levels and role of ZNF703 in NSCLC. Our results indicate that ZNF703 is a new candidate gene in lung cancer and may contribute to the development of lung tumors by activating the Akt/mTOR pathway.

## Conflict of Interest

The authors declare that they have no competing interests.

## Supporting information


**Figure S1.** Representative Photographs of the Western Blots. (A) p‐Akt and (B) Akt.

**Table S1.** Intensities of Akt1 and p‐Akt1 obtained by Image Analysis.
