# Supplementary figures and images for: ZNF703 Overexpression may act as an oncogene in non‐small cell lung cancer
Source: Cancer Med. 2016 Sep 20;5(10):2873–8. doi: 10.1002/cam4.847 (PMC5083741; doi:10.1002/cam4.847)

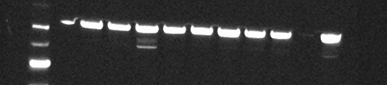


A.


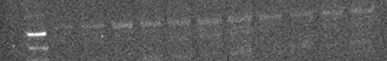


B.

Supplementary Figure 1: Representative Photographs of the Western Blots. A. p-Akt and B. Akt

Supplement: Supplementary file 1 — Figure S1. Representative Photographs of the Western Blots. (A) p‐Akt and (B) Akt. [file CAM4-5-2873-s001.docx]
